# Preclinical Evaluation of Anticancer Efficacy and Pharmacological Properties of FBA-TPQ, a Novel Synthetic Makaluvamine Analog

**DOI:** 10.3390/md10051138

**Published:** 2012-05-23

**Authors:** Xiangrong Zhang, Hongxia Xu, Xu Zhang, Sukesh Voruganti, Srinivasan Murugesan, Dwayaja H. Nadkarni, Sadanandan E. Velu, Ming-Hai Wang, Wei Wang, Ruiwen Zhang

**Affiliations:** 1 Department of Pharmaceutical Sciences, School of Pharmacy, Texas Tech University Health Sciences Center, Amarillo, TX 79106, USA; Email: zhangxr@vip.sina.com (X.Z.); red_clara@yahoo.com.cn (H.X.); xu.zhang@ttuhsc.edu (X.Z.); sukesh.voruganti@ttuhsc.edu (S.V.); 2 Department of Chemistry, University of Alabama at Birmingham, Birmingham, AL 35294, USA; Email: murugesan.srinivasan@gmail.com (S.M.); dwayaja@gmail.com (D.H.N.); svelu@uab.edu (S.E.V.); 3 Cancer Biology Center, School of Pharmacy, Texas Tech University Health Sciences Center, Amarillo, TX 79106, USA; Email: MingHai.Wang@ttuhsc.edu; 4 Department of Biomedical Sciences, Texas Tech University Health Sciences Center, Amarillo, TX 79106, USA

**Keywords:** pancreatic cancer, marine anticancer agents, RRLC, pharmacokinetics

## Abstract

We have recently designed and synthesized a novel iminoquinone anticancer agent, 7-(4-fluorobenzylamino)-1,3,4,8-tetrahydropyrrolo[4,3,2-de]quinolin-8(1*H*)-one (FBA-TPQ) and initiated its preclinical development. Herein we investigated its efficacy, safety, and pharmacokinetics in *in vitro* and *in vivo* models of human pancreatic cancer. Our results demonstrated that FBA-TPQ inhibited pancreatic cancer cell growth, induced apoptosis, and caused cell cycle arrest *in vitro*. It inhibited the growth of xenograft tumors with minimal host toxicity. To facilitate future preclinical and clinical development of the agent, we also developed and validated a Rapid Resolution Liquid Chromatography (RRLC) method for quantitative analysis of FBA-TPQ in plasma and tissue samples. The method was found to be precise, accurate, and specific. Using this method, we carried out *in vitro* and *in vivo* evaluations of the pharmacological properties of FBA-TPQ, including stability in plasma, plasma protein binding, metabolism by S9 enzymes, plasma pharmacokinetics, and tissue distribution. Our results indicate that FBA-TPQ is a potential therapeutic agent for pancreatic cancer, providing a basis for future preclinical and clinical development.

## 1. Introduction

Cancer remains a major public health problem despite the introduction of novel diagnostic procedures, improved surgical techniques and new radiation therapy technologies, alongside the development of targeted therapies. While the prognosis of many cancers is much better than a few decades ago, many cancers are still difficult to treat, especially pancreatic cancer, one of the most deadly of all cancers [1]. Standard therapy for pancreatic cancer is multimodal, involving surgery and chemotherapy. Since clinical symptoms are often non-specific and consequently most patients are diagnosed at advanced stages without any surgical options, chemotherapy becomes a limited choice of treatment. Thus far, gemcitabine has remained the single first-line chemotherapeutic agent for advanced pancreatic cancer; however, less than 25% of patients benefit from this drug [2]. Therefore, there is an urgent need to develop more effective and safer chemotherapeutic agents for pancreatic cancer. While the recently developed targeted therapies, such as trastuzumab, erlotinib, and imatinib, have improved the treatment outcomes of certain patients with cancer, new broad-spectrum chemotherapeutic agents with lower toxicity than the existing agents are still needed to achieve complete tumor eradication, to prevent recurrence, or to better prepare patients for curative resection. Additionally, such broad-spectrum agents may be useful in combination with targeted therapies to ensure the eradication of all cells, decreasing the likelihood of the development of acquired resistance. 

The search for new lead compounds is a crucial element in anticancer drug discovery. Natural products have historically provided many successful new anti-cancer drugs (e.g., doxorubicin, etoposide, paclitaxel, and gemcitabine). One class of the rich resources is marine products that have provided a large number of anticancer agents with novel structures [3,4,5,6,7,8,9]. To our best knowledge, cytarabine is the only US Food and Drug Administration (FDA)-approved marine-derived anticancer drug in the US Pharmacopeia. Trabectedin has been approved by the European Agency for the Evaluation of Medicinal Products (EMEA), and is under Phase III studies in the US. Examples of new experimental anticancer agents derived from marine natural products that have entered pre-clinical and clinical trials within the last decade include eribulin mesylate, soblidotin, plitidepsin, elisidepsin, PM1004, tasidotin and hemiasterlin [10]. 

**Figure 1:**
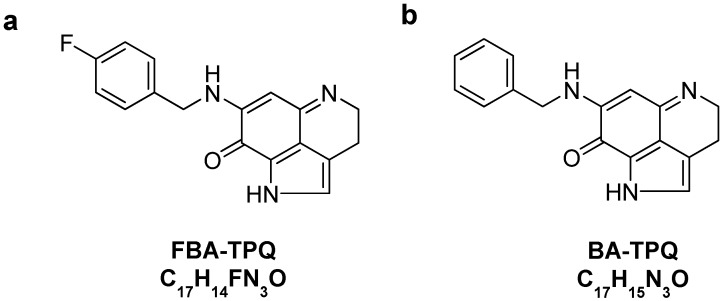
The structures of FBA-TPQ (**a**) and BA-TPQ (**b**) internal standard.

Makaluvamines, a class of marine iminoquinone alkaloids isolated from sponges of the genera *Zyzzya*, have been reported to have potent *in vitro* and *in vivo* cytotoxicity against several human cancer cell lines [10,11,12]. We have recently designed and synthesized several makaluvamine analogs and demonstrated their anti-cancer activities and safety in *in vitro* and *in vivo* tumor models [11,12,13,14,15]. 7-(4-fluorobenzylamino)-1,3,4,8-tetrahydropyrrolo[4,3,2-de]quinolin-8(1*H*)-one (FBA-TPQ; Figure 1a one of the most effective compounds in the class has been shown to increase apoptosis, inhibit proliferation and decrease cell viability of prostate [13], breast [14] and ovarian cancer cells [15], and to decrease the growth of breast and ovarian cancer xenograft tumors, with an acceptable safety profile [14,15]. In the present report, we evaluated the anti-tumor activities of FBA-TPQ against pancreatic cancer cells. As part of the continuing development of this compound, we developed a rapid resolution liquid chromatography (RRLC) method for FBA-TPQ. We validated the new RRLC method for various biological matrices, and examined the *in vitro* and *in vivo* pharmacological properties of FBA-TPQ, following intravenous and intraperitoneal administrations to BALB/c mice. We believe that the results presented here would provide a basis for future preclinical and clinical development of this compound. 

## 2. Results and Discussion

### 2.1. FBA-TPQ Exerts *in Vitro* Anticancer Activity against Pancreatic Cancer Cells

#### 2.1.1. Inhibition of Cancer Cell Growth

**Figure 2:**
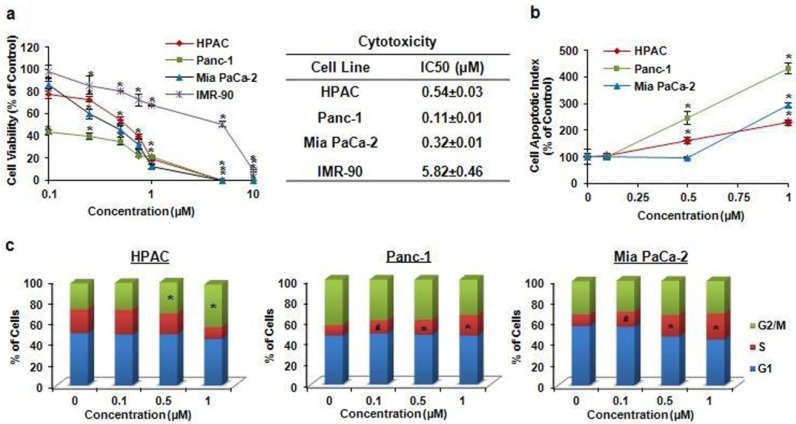
(**a**) Cell growth inhibitory activity of FBA-TPQin human pancreatic cancer cells and primary fibroblasts. HPAC, Panc-1, Mia PaCa-2 and IMR-90 cells were exposed to various concentrations of FBA-TPQfor 72 h, followed by MTT assay; (**b**) Induction of apoptosis in pancreatic cancer cells by FBA-TPQ. HPAC, Panc-1, Mia PaCa-2 cells were exposed to various concentrations of the compound for 48 h, followed by measurement of apoptosis by Annexin V assay/flow cytometry. The apoptotic index was calculated against untreated control cells; (**c**) Cell cycle progression effect of FBA-TPQon human pancreatic cancer cells. Cells were exposed to various concentrations of the compound for 48 h, followed by determination of cell cycle distribution. All assays were performed in triplicate. (^#^*P* < 0.05, * *P* < 0.01).

We evaluated the effects of FBA-TPQ on the survival of several human pancreatic cancer cell lines, including HPAC (p53^+/+^), Panc-1(p53^+/−^), and Mia PaCa-2(p53^+/−^), as well as normal IRM90 fetal fibroblast cells (Figure 2a). Cells were exposed to various concentrations of the test compound (0–10 µM) for 72 h, and cell survival rates were determined by the MTT assay, using a procedure reported previously [14,16,17]. FBA-TPQ exerted potent effects against the test cancer cell lines, leading to significant decreases in cell viability. As shown in Figure 2a the compound demonstrated IC_50_ (the concentration that inhibits the survival of cells by 50%) values of less than 1 μM (0.11–0.54 μM); normal IMR90 fibroblasts were significantly less sensitive to the inhibitory effects of FBA-TPQ than the pancreatic cancer cells, with 10–50-fold differences in IC_50_, indicating the specificity of the compound. 

#### 2.1.2. Induction of Apoptosis

The apoptotic cells were detected using a method reported previously [14,18,19]. As illustrated in Figure 2b, FBA-TPQ induced apoptosis in a dose-dependent manner in all three cell lines. In HPAC cells, a 1 µM concentration of FBA-TPQ increased the apoptotic index two-fold higher than that seen in control cells (*P* < 0.01). In Panc-1 cells, FBA-TPQ at 1 µM demonstrated a four-fold increase in apoptosis (*P* < 0.01). In the Mia PaCa-2 cells, FBA-TPQ at 1 µM led to a three-fold increase in apoptosis (*P* < 0.01). Although both of HPAC and Panc-1 cells showed a significant increase in apoptosis beginning at the 0.5 µM concentration (*P* < 0.01), the Panc-1 cells were significantly more sensitive than the HPAC cells (Figure 2b). 

#### 2.1.3. Cell Cycle Arrest

The effects of FBA-TPQ on cell cycle distribution were analyzed using the previously reported methods [20,21]; its effects appeared to be cell-line-dependent (Figure 2c). At 1 µM, FBA-TPQ induced an arrest in the G2/M phase (*P* < 0.01) in HPAC cells; in Panc-1 and Mia PaCa-2 cells, it induced arrest in the S phase (*P* < 0.01). The differences in the responses of the different cell lines may be related to their expression of p53.

### 2.2. FBA-TPQ Decreases the Growth of Xenograft Tumors

**Figure 3:**
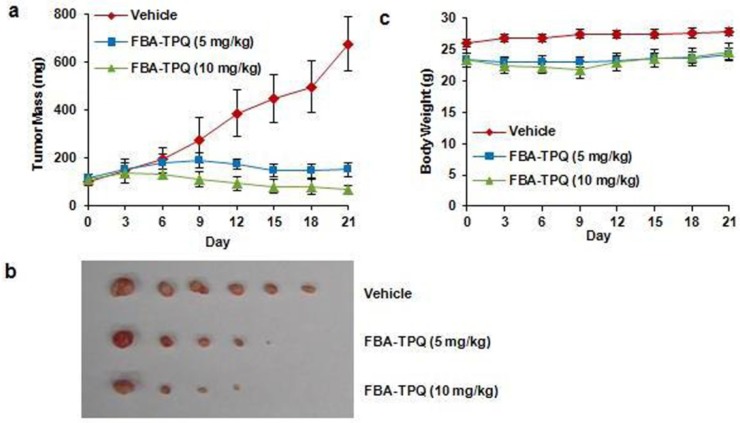
*In vivo* effects of FBA-TPQ administered to nude mice bearing Panc-1 xenograft tumors. (**a**) FBA-TPQ was administered by intraperitoneal (ip) injection at doses of 5 mg/kg/day, 5 days/week for 3 weeks and 10 mg/kg/day, 5 days/week for 2 weeks, tumors were measured every three days; (**b**) At the end of the experiment, representative tumors were removed and photographed; (**c**) Animals were also monitored for changes in body weight as a surrogate marker for toxicity.

Since it exerted potent effects *in vitro*, we determined whether FBA-TPQ could also show anti-tumor effects *in vivo*. Nude mice bearing Panc-1 xenograft tumors were treated with vehicle or FBA-TPQ at 5 or 10 mg/kg/day, 5 days per week. Compared with control group, treatments of 5 and 10 mg/kg/day resulted in 77.8 (*P* < 0.01) and 90.1% (*P* < 0.01) inhibition of tumor growth, respectively (Figure 3a,b), with significant tumor regression or complete remission being seen in both treated groups. No significant host toxicity (using body weight as a surrogate marker) was observed at any of the doses (Figure 3c), suggesting that the FBA-TPQ can be safely given as a novel therapeutic agent.

Previous studies have shown that FBA-TPQ has nanomolar/low micromolar IC_50_ values against prostate, breast, and ovarian cancer cell lines [13,14,15]. The compound inhibited cell growth, decreased cell proliferation, induced apoptosis, and arrested cell cycle distribution. In addition, normal cells were not as sensitive to the compound. *In vivo* study demonstrated that FBA-TPQ has potent activity against breast and ovarian cancer xenograft tumors [14,15]. Mechanistic studies revealed that FBA-TPQ down-regulated MDM2, E2F1, Bcl-2, PI3K proteins, up-regulated Fas, Bax, p53/p-p53, ATM/p-ATM, γH2AX, and led to increased cleavage of caspases-3, -8 and -9 both *in vitro* and *in vivo* [13,14,15]. These findings suggested that FBA-TPQ has potent anti-cancer activity due to inhibition of oncogenes, activation of the DNA damage response and PI3K-Akt pathways. In summary, our preclinical efficacy and safety data indicate that FBA-TPQ can be a candidate anticancer drug for future development. In subsequent studies, we developed analytical methods and determined pharmacological properties of the compound, including initial pharmacokinetic and metabolic studies. 

### 2.3. RRLC Method Development and Validation

The chromatographic conditions, such as the mobile phase composition, elution gradients, and column temperature, were optimized during the development of the RRLC methods. Acetonitrile was chosen over methanol for its ability to reduce back-pressure and achieve better separation. BA-TPQ (Figure 1b) was chosen as the internal standard due to its similarity to FBA-TPQ in terms of extractability and chromatographic behavior [11,12,22,23].

#### 2.3.1. Chromatographic Separation and UV Detection of FBA-TPQ in Different Matrices

**Figure 4:**
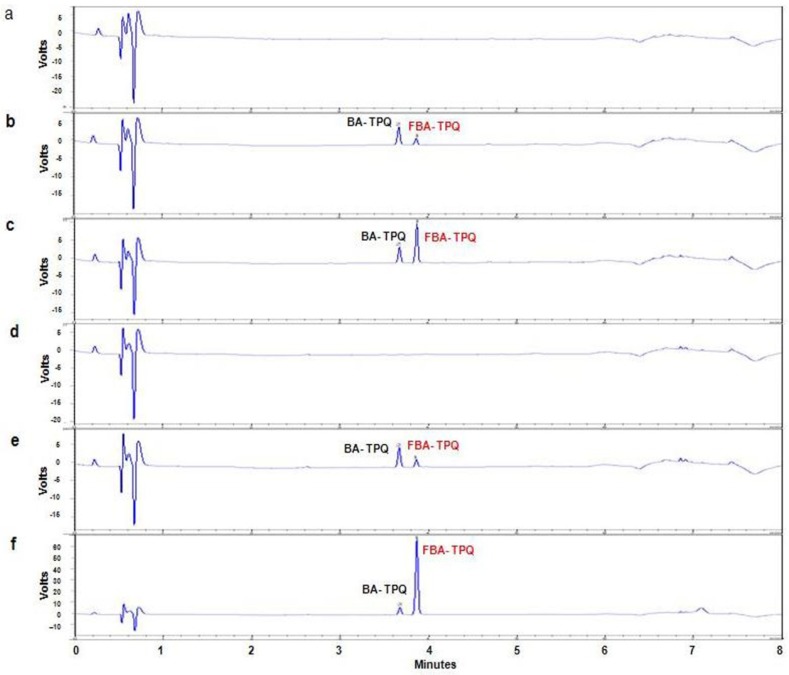
Chromatograms of FBA-TPQ obtained using the optimized method. (**a**) A blank mouse plasma sample; (**b**) A mouse plasma sample spiked with 0.5 µM FBA-TQP; (**c**) A plasma sample collected 0.08 h after intravenous (iv) administration of (Dose = 5 mg/kg) FBA-TPQ; (**d**) A blank lung sample; (**e**) A lung sample spiked with FBA-TPQ at 0.5 µM; (**f**) A lung sample collected 0.08 h after iv injection of FBA-TPQ.

The specificity of the RRLC assay was examined using the chromatographic conditions described in the *Experimental* section. Representative chromatograms of blank mouse plasma, control mouse plasma spiked with 0.5 µM FBA-TPQ, and a plasma sample obtained from a BALB/c mouse five minutes after intravenous administration of 5 mg/kg are shown in Figure 4a–c. Representative chromatograms of blank lung tissue, a control lung tissue sample spiked with 0.5 µM FBA-TPQ, and a lung tissue sample obtained from a BALB/c mouse five minutes after intravenous administration of 5 mg/kg are shown in Figure 4d–f. Similar chromatograms were obtained for the other tissues examined. The specificity was demonstrated by the absence of any endogenous interference in the biological samples at the retention time of the peaks of FBA-TPQ and internal standard (BA-TPQ). 

#### 2.3.2. Linearity of the Calibration Curve, Lower Limit of Detection, and Lower Limit of Quantitation

Calibration curves (peak area ratio of the analyte derivative to the IS derivative *versus* the nominal analyte concentration) were fitted by weighted (1/*x*^2^) least-square linear regression. The correlation coefficient of the calibration curves for FBA-TPQ in plasma and various tissues were all above 0.9937. All of the linearity and range parameters are shown in Table 1, along with the associated validation data. 

**Table 1:** FBA-TPQ Standard Curve Calculation by Least Squares Regression Analysis.

Tissue	LOD (µM)	LOQ (µM)	Linear Fit	Correlation Coefficient ( *R*)	Linear Range (µM)
Plasma	0.010	0.050	*Y* = 0.7206*x* + 0.0015	0.9963	0.050–10
Liver	0.020	0.050	*Y* = 0.7465*x* + 0.0370	0.9937	0.050–10
Brain	0.020	0.050	*Y* = 0.6637*x* + 0.0238	0.9989	0.050–10
Spleen	0.020	0.050	*Y* = 0.7810*x* + 0.0018	0.9964	0.050–10
Heart	0.020	0.050	*Y* = 0.7242*x* + 0.0183	0.9965	0.050–10
Lungs	0.020	0.050	*Y* = 0.6707*x* + 0.0016	0.9946	0.050–20
Kidneys	0.020	0.050	*Y* = 0.7281*x* + 0.0036	0.9993	0.050–20

The lower limits of detection (LLOD) and lower limits of quantitation (LLOQ) for FBA-TPQ using the optimized conditions were 0.01 and 0.05 μM, respectively in mouse plasma. The coefficient of variation (CV) was 5.04% and the accuracy was 118.52 ± 3.4% at the LLOQ.

#### 2.3.3. Precision and Accuracy

Table 2 shows the intra- and inter-day precision and accuracy for the detection of FBA-TPQ in mouse plasma and tissues using the RRLC method. The accuracy was defined as the percentage of the concentration that was present after being calculated from peak areas compared to the known concentration of prepared samples. Precision was defined as the variation between replicate samples. The intra- and inter-day coefficients of variation for FBA-TPQ were <5.41% and <11.4%, respectively. The accuracy of the quantitative analysis of the compound ranged from 91.0 to 119.0% for the intra-day and 93.73 to 116.0% for the inter-day analyses. Both the precision and accuracy were well within the acceptable range as described by the FDA [24]. 

**Table 2:** The accuracy and precision of the FBA-TPQ RRLC method in various tissue samples.

Tissue	Theoretical Concentration (µM)	Intra-Day Assay (*n* = 5)			Inter-Day Assay (*n* = 4)		
		Measured Concentration (µM)	RSD (%)	Accuracy (%)	Measured Concentration (µM)	RSD (%)	Accuracy (%)
Plasma	0.100	0.100 ± 0.005	5.41	100	0.100 ± 0.010	11.4	98.7
	2.00	2.03 ± 0.058	2.86	101	2.07 ± 0.112	5.41	100
	10.0	10.3 ± 0.026	0.251	103	9.94 ± 1.46	14.6	99.4
Liver	0.100	0.100 ± 0.010	12.7	99.7	0.103 ± 0.010	9.56	103
	2.00	2.01 ± 0.010	0.440	100	2.03 ± 0.010	0.550	101
	10.0	4.99 ± 0.089	0.570	102	10.2 ± 0.030	0.310	102
Brain	0.100	0.0510 ± 0.004	14.2	101	0.096 ± 0.013	13.1	95.7
	2.00	0.522 ± 0.038	1.19	104	2.06 ± 0.022	1.06	103
	10.0	4.80 ± 0.199	3.62	96.0	9.79 ± 0.410	4.19	97.9
Spleen	0.100	0.101 ± 0.001	0.571	100	0.101 ± 0.001	0.990	101
	2.00	1.99 ± 0.023	0.330	98.5	1.97 ± 0.007	1.18	99.5
	10.0	10.3 ± 0.176	0.708	102	10.2 ± 0.073	1.71	102
Heart	0.100	0.100 ± 0.001	1.00	100	0.100 ± 0.001	0.580	99.7
	2.00	1.93 ± 0.025	1.27	96.4	1.96 ± 0.022	1.13	98.0
	10.0	10.4 ± 0.267	2.58	103	10.1 ± 0.070	0.700	101
Lungs	0.100	0.0910 ± 0.008	8.79	91.0	0.0920 ± 0.001	1.09	92.0
	5.00	4.57 ± 0.114	2.50	91.3	4.69 ± 0.106	2.28	93.7
	20.0	23.0 ± 0.008	0.0300	115	23.0 ± 0.056	0.250	114
Kidneys	0.100	0.119 ± 0.001	0.841	119	0.116 ± 0.007	5.97	116
	5.00	4.85 ± 0.075	1.54	97.1	4.84 ± 0.057	1.17	96.8
	20.0	21.8 ± 0.083	0.382	109	21.8 ± 0.026	0.120	109

#### 2.3.4. Extraction Recovery

The extraction recoveries of the compound were determined at low (0.1 μM), medium (2.0 μM), and high (10 μM) concentrations (in triplicate) in plasma, and in spleen, liver, brain and heart homogenates, and at low (0.1 μM), medium (5.0 μM), and high (20 μM) concentrations (in triplicate) in lung and kidney homogenates. The recovery percentages for the compound extracted from mouse plasma and various tissues at the three concentrations are shown in Table 3. In general, the recovery at lower concentration (0.1 μM) was lower than that of higher concentrations. However, these differences would not affect the standard curve preparation and the results of drug analysis of the biological samples, due to the use of the internal standard and the same procedures used in the preparation of the standard curves and the actual biological samples.

**Table 3:** Recovery of FBA-TPQ from various tissue homogenates.

Tissue	Concentration (μM)	Recovery (%) ± SD (*n* = 5)	RSD (%)
Plasma	0.100	83.6 ± 0.71	0.991
	5.00	95.3 ± 1.36	1.55
	10.0	90.9 ± 2.72	2.91
Liver	0.100	77.5 ± 3.99	5.58
	5.00	87.8 ± 4.48	5.96
	10.0	94.8 ± 2.58	2.99
Brain	0.10	72.4 ± 3.21	4.39
	5.00	82.4 ± 0.96	1.17
	10.0	89.7 ± 0.58	0.66
Spleen	0.100	75.3 ± 2.34	3.16
	5.00	91.9 ± 2.06	2.41
	10.0	95.1 ± 2.00	2.28
Heart	0.100	83.2 ± 0.71	1.75
	5.00	93.2 ± 1.36	2.20
	10.0	96.4 ± 2.72	0.210
Lungs	0.100	75.7 ± 1.32	5.62
	5.00	86.3 ± 1.72	0.320
	20.0	85.7 ± 0.17	0.84
Kidneys	0.100	75. 9 ± 2.75	3.74
	5.00	88.8 ± 1.24	1.58
	20.0	95.5 ± 1.54	1.86

#### 2.3.5. Stability

The stability experiments showed that FBA-TPQ was stable for 24 h after preparation at 22 °C, for 2 h at 22 °C following three freeze/thaw cycles (−20 to 22 °C) on consecutive days, and for 3 months at −80 °C, as the RE values were within ±15% for both the low and high concentrations. Taken together, the stability data indicated that FBA-TPQ in plasma and tissue samples could be stored and prepared under routine laboratory conditions.

### 2.4. Protein Binding

To determine the extent to which FBA-TPQ is bound by plasma proteins, pooled plasma samples were examined for their capacity to bind the compound at concentrations of 0.5 μM and 5 μM. FBA-TPQ was considerably bound to proteins in mouse plasma, with 72.32% ± 2.53%; and 77.78% ± 1.17% of the compound being bound to plasma proteins at concentrations of 0.5 μM and 5.0 μM, respectively. Extensive binding of a candidate compound to plasma proteins can impair its biodistribution and may therefore impact its therapeutic efficacy. However, compounds that are highly bound to proteins display longer half-lives, durations of action, and higher volumes of distribution. In the present study, FBA-TPQ exhibited considerable protein binding, which may have limited the *in vitro* degradation of the compound, as described below. 

### 2.5. Metabolism by Mouse S9 Enzymes

As a preliminary study of the metabolism of FBA-TPQ, the compound was incubated with S9 fractions containing Phase I and Phase II metabolic enzymes and their co-factors for 15, 30, 45, or 60 min. These studies indicated that FBA-TPQ undergoes significant metabolism in the presence of both Phase I and Phase II enzymatic components, with approximately 14.3% of the compound being metabolized by Phase I and 1.8% of the compound being metabolized by Phase II components during the 60 min incubation. No specific peaks that seemed to correspond to unique metabolites were identified using the current RRLC method.

### 2.6. Pharmacokinetics of FBA-TPQ in Mice

**Figure 5:**
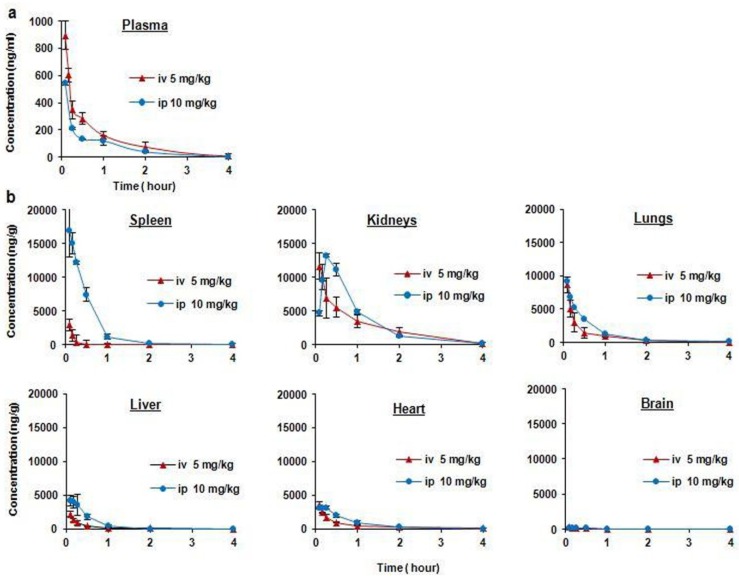
(**a**) Mean plasma concentration-time curves of FBA-TPQ after intravenous and intraperitoneal injection in BALB/c mice (mean±SD); (**b**) Mean tissue concentration-time curves of FBA-TPQ after intravenous and intraperitoneal injection in BALB/c mice (mean±SD).

To assess the disposition of FBA-TPQ *in vivo*, pharmacokinetic studies in BALB/c were conducted. The results of the mean plasma concentration-time curves of FBA-TPQ, and tissue concentration-time curves of FBA-TPQ, after intravenous or intraperitoneal injection in BALB/c mice are presented in Figure 5. Two-compartmental model (WinNonlin 6.0 Model 7, Pharsight Corporation: Mountain View, CA, USA, 2009) was used to analyze the data obtained following intravenous (iv) and intraperitoneal (ip) administration of the compound. The pharmacokinetic parameters are presented in Table 4.

After a single bolus intravenous dose of FBA-TPQ (5 mg/kg), there were relatively high concentrations of the compound in the plasma for the first 30 minutes, with a rapid decline thereafter, as indicated by its half-life of 0.248 h, and clearance rate of 8.46 (L/hr/kg) in BALB/c mice (Table 4). The iminoquinone was no longer detectable in mouse plasma 4 h after the injection. The highest overall concentrations in the tissues examined were in the order kidneys > lungs > heart > spleen > plasma ≈ liver > brain.

**Table 4:** Pharmacokinetic parameters for FBA-TPQ following intravenous or intraperitoneal injection in BALB/c mice.

Tissue	Route	Dose (mg/kg)	T_1/2_ (h)		AUC (h·µg/mL)	Cl (L/h/kg)	V_ss_ (mL/g)
Plasma	iv	5	0.248		0.583	8.46	18.7
	ip	10	0.219		0.338	19.8	21.8
Liver	iv	5	0.510		0.949	4.70	3.34
	ip	10	0.127		1.85	3.85	6,72
Brain	iv	5	0.252		0.138	34.5	18.7
	ip	10	1.83		0.0623	2.13	4.10
Spleen	iv	5	0.081	2.33	1.98	1.27
	ip	10	1.73	6.50	0.932	0.78
Heart	iv	5	0.502	2.00	2.47	1.77
	ip	10	0.541	2.36	3.85	2.19
Lungs	iv	5	0.501	4.02	1.237	7.79
	ip	10	0.363	5.71	1.88	10.32
Kidneys	iv	5	0.251	11.5	0.436	0.491
	ip	10	1.58	14.7	0.823	0.801

T_1/2_: half-life of the compound; AUC: area under the concentration-time curve; Cl: clearance; Vss: volume in steady state.

Following intraperitoneal administration, the concentration of the compound in plasma decreased rapidly from 542.5 ng/mL at 5 min to 40.25 ng/mL at 2 h, to undetectable levels 4 h after administration. The highest overall tissue concentrations after intraperitoneal injection were in the order: kidneys > spleen > lungs > liver > heart > brain. The intraperitoneal bioavailability of FBA-TPQ in BALB/c mice was estimated to be 28.91%, based on the AUC values.

The pharmacokinetic profiles for the tissues obtained in our study suggest that there is high drug accumulation in the lungs, kidneys, and spleen of the mice, and that it reaches low concentrations in the brain. Furthermore, pharmacokinetic modeling confirmed that these tissues exhibited the highest drug exposure (AUC), longest half-life, and lowest clearance values, indicating that these organs may be potential sites of FBA-TPQ toxicity.

Our study also investigated the pharmacokinetic profile of FBA-TPQ following both intravenous (5 mg/kg) and intraperitoneal (10 mg/kg) administration. Based on the AUC values, the bioavailability of FBA-TPQ following intraperitoneal injection was relatively low, presumably because FBA-TPQ underwent extensive metabolism after ip injection and/or distributed in tissues quickly. Ultimately, it would be optimal if the compound could be administered orally, because patients greatly prefer oral agents. Studies are currently underway to develop a method for oral delivery of the compound.

## 3. Experimental Section

### 3.1. Test Compound, Chemicals and Reagents

FBA-TPQ and BA-TPQ (Figure 1a,b) were kindly provided by Dr. Sadanandan Velu (University of Alabama at Birmingham). Acetonitrile and methanol were purchased from Fisher Scientific (Atlanta, GA, USA). All other chemicals were purchased from Sigma Chemical Company (St. Louis, MO, USA). Heparinized samples of non-Swiss albino mouse plasma were obtained from Lampire Biological Laboratories (Pipersville, PA, USA). Hepatic S9 fractions (20 mg/mL) from male ICR/CD-1 mice were purchased from Celsis In Vitro Technologies (Chicago, IL, USA). All chemicals and solvents used for sample preparation and the rapid resolution liquid chromatography analysis were of analytical grade. Cell culture supplies and media, phosphate-buffered saline (PBS), fetal bovine serum (FBS), sodium pyruvate, non-essential amino acids, and penicillin-streptomycin were obtained from Invitrogen (Carlsbad, CA, USA).

### 3.2. Cell Culture

Human pancreatic cancer cells were obtained from the American Type Culture Collection (Rockville, MD). All cell culture media contained 10% FBS and 1% penicillin/streptomycin. HPAC cells were grown in a 1:1 mixture of Dulbecco’s modified Eagle’s medium and Ham’s F12 medium containing 1.2 g/L sodium bicarbonate, 2.5 mM L-glutamine, 15 mM HEPES and 0.5 mM sodium pyruvate supplemented with 2 µg/mL insulin, 5 µg/mL transferrin, 40 ng/mL hydrocortisone, 10 ng/mL epidermal growth factor and 5% fetal bovine serum. Panc-1 cells were cultured with RPMI 1640 containing 1 mM HEPES buffer, 25 µg/mL gentamicin, 1.5 g/L sodium bicarbonate, and 0.25 µg/mL amphotericin B. Mia PaCa-2 and IMR-90 cells were grown in DMEM media. 

### 3.3. MTT Cell Survival Assay

The effect of the test compound on human pancreatic cancer cell viability, expressed as the percentage of cell survival, was determined using the MTT (3-(4,5-dimethylthiazol-2-yl)-2,5-diphenyl tetrazolium bromide) assay. The MTT assay is colorimetric assay for measuring the activity of enzymes that reduce MTT to formazan dyes, giving a purple color. Cells were grown in 96-well plates at 4–5 × 10^3^ cells per well and exposed to different concentrations of the test compound (0, 0.1, 0.25, 0.5, 0.75, 1, 5 and 10 µM). After incubation for different times, 10 µL of the MTT solution (5 mg/mL; Sigma; St. Louis, MO, USA) were added into each well. The plates were incubated for 2–4 h at 37 °C. The supernatant was then removed and the formazan crystals were dissolved with 100 µL of DMSO. The absorbance at 570 nm was recorded using an OPTImax microplate reader (Molecular Devices; Sunnyvale, CA, USA). The cell survival percentages were calculated by dividing the mean OD of compound-containing wells by that of DMSO-containing control wells. Three separate experiments were accomplished to determine the IC_50_ values.

### 3.4. Detection of Apoptosis

Following a similar protocol as above, cells in early and late stages of apoptosis were detected using an Annexin V-FITC apoptosis detection kit from BioVision (Mountain View, CA, USA), according to the manufacturer’s protocol. For apoptosis experiments, 2–3 × 10^5^ cells were exposed to the test compound (0, 0.1, 0.5 and 1.0 µM) and incubated for 48 h prior to analysis. Cells were collected and washed with serum-free media. Cells were then re-suspended in 500 µL of Annexin V binding buffer followed by addition of 5 µL of Annexin V-FITC and 5 µL of propidium iodide (PI). The samples were incubated in the dark for 5 min at room temperature and analyzed with a Becton Dickinson FACSCalibur instrument (Ex = 488 nm; Em = 530 nm). Cells that were positive for Annexin V-FITC alone (early apoptosis) and Annexin V-FITC and PI (late apoptosis) were counted. The apoptotic index was calculated against the untreated control cells.

### 3.5. Cell Cycle Measurements

To determine the effect of FBA-TPQ on the cell cycle, cells (2–3 × 10^5^) were exposed to the test compounds (0, 0.1, 0.5 or 1 µM) and incubated for 24 h prior to analysis. Cells were trypsinized, washed with PBS, and fixed in 1.5 mL of 95% ethanol and 0.5 mL of 0.9% saline solution at 4 °C overnight, followed by incubation with RNAse and staining with propidium iodide (Sigma). The DNA content was determined by flow cytometry. The cell cycle distribution was evaluated by comparing with that of the control cells. 

### 3.6. Instruments and Conditions

An Agilent 1200 Series Rapid Resolution Liquid Chromatography system (Agilent Technologies, Waldbronn, Germany) consisted of an on-line degaser, a binary pump, a high performance SL autosampler, a thermostated column compartment, and a photodiode array UV-vis detector. Determination of FBA-TPQ was achieved using a Zorbax XDB-C_18_ (1.8 μm, 50 × 4.6 mm) analytical column with a LiChroCART 100 RP-18 guard column. The column eluate was monitored by a diode array detector at 354 nm. The temperature was maintained at 35 °C. The system was set to use a linear gradient at a flow rate of 0.9 mL/min, increasing the acetonitrile-water ratio from 10:90 at 0 min to 38:62 at 3.5 min, keeping this ratio until 5 min, and then increasing to 85:15 in 0.1 min, maintaining this ratio for 1 min, and then returning to 10: 90 in 0.4 min, for a total running time of 8 min. 

### 3.7. Preparation of Calibration Standards and Quality Control Samples

A 1 mM stock solution of FBA-TPQ was prepared in methanol and stored at −80 °C. A series of standard working solutions with FBA-TPQ concentrations of 0.1, 0.25, 0.5, 1.0, 5.0, 10.0, 50.0 and 100.0 μM were obtained by further dilution of the stock solution with methanol. The standard serum samples for calibration were prepared at concentrations of 0.1, 0.25, 0.5, 1.0, 5.0, and 10.0 μM in control (drug-free) pooled mouse plasma. 

Calibration curves were prepared by spiking 20 μL of the appropriate standard solution and 20 μL BA-TPQ (IS) into 160 μL of blank mouse plasma. The 40 µM BA-TPQ solution was prepared by diluting a stock solution of 100 µM BA-TPQ with methanol. Quality control samples of low, medium and high concentrations were prepared to assess the accuracy and precision of the bioanalytical method. For the calibration of the tissue samples, a plasma sample was supplemented with tissue homogenates in PBS. 

### 3.8. Sample Preparation

Acetonitrile (600 μL) and BA-TPQ (IS, 20 μL) were added to 200 μL of plasma to precipitate proteins. The mixture was vigorously vortexed for 1 min and centrifuged at 14,000 × g for 10 min. Then, the supernatant was transferred to a glass tube and evaporated to dryness at 40 °C under a stream of nitrogen in a TurboVap^®^ LV Concentration Workstation (Caliper Lifesciences, Hopkinton, MA, USA). The resulting supernatant was transferred to a new glass tube and evaporated to dryness. Finally, samples were reconstituted in 200 μL mobile phase, and 10 μL was injected into the RRLC system for analysis.

### 3.9. Method Validation

To confirm its accuracy, selectivity, reproducibility, and specificity, the method was validated as follows: Evaluation of the method selectivity was performed by analyzing the blank plasma from six different preparations to test for interference at the retention times of the analyte. The linearity of the method was determined by plotting the peak area ratios of the analyte to the internal standard (IS) against the concentrations of FBA-TPQ in mouse plasma and various tissues. This was done in duplicate on three consecutive validation days. The precision and accuracy of the method were assessed by the determination of QC samples at three concentration levels, in six replicates, on three validation days. For the intra- and inter-day precision, we required that there was less than 15% variation, and also required the accuracy to be within ±15%, which is within the limits proposed in the US FDA guidelines [24]. Limit of detection (LOD) was the lowest concentration of an analyte that the bioanalytical procedure can reliably differentiate from background noise. The LLOQ, defined as the lowest concentration on the calibration curve at which both precision and accuracy were less than or equal to 20%, was evaluated by analyzing samples prepared in six replicates on three consecutive validation days [24]. The recovery of FBA-TPQ was determined by comparing the mean peak areas of the pretreated QC samples at three concentration levels (six samples each) to those of spike-after-extraction samples.

### 3.10. Stability in Plasma

The stability of FBA-TPQ was evaluated at two concentrations (0.5 and 5.0 µM). The compound was incubated under the following conditions: 37 °C for 0, 1, 2, 4, and 8 h; 4 °C for 0, 1, 4, 8, and 24 h; and −80 °C for 0, 1, 2, 4, and 12 weeks. At the designated time points, the samples were extracted and analyzed using the established RRLC analytical method. The stability of FBA-TPQ was assessed by comparing the initial concentration (time 0) with the final concentration following incubation of the compound at the noted temperatures, and expressing this difference as a percentage of the initial concentration.

### 3.11. Binding to Plasma

The extent of binding to mouse plasma proteins by FBA-TPQ was determined using a micro-ultrafiltration system as previously described [25]. FBA-TPQ was dissolved in methanol and added to pooled mouse plasma to yield final concentrations of 0.5 µM and 5.0 µM. Control solutions were prepared in methanol to account for non-specific binding of FBA-TPQ to the filter membranes. Duplicate preparations of each concentration were incubated at 37 °C for 1 h, before being placed into the sample reservoirs of Amicon Centrifree^®^ ultrafiltration tubes (30 kDa exclusion; Millipore).

### 3.12. S9 Metabolism

A preliminary study of the metabolism of FBA-TPQ was done using murine hepatic S9 fractions containing Phase I and II metabolic enzymes. The reaction mixture contained 10 μM FBA-TPQ (in methanol), 1 mg/mL S9 and 100 mM Tris buffer (pH 7.4) (experimental controls did not contain the hepatic S9 fractions.) Metabolic reactions were initiated by adding NADPH-regenerating systems to the reaction mixtures, and samples were incubated in a water bath at 37 °C. Duplicate aliquots of the mixtures were taken at 0, 15, 30, 45, and 60 min, and the samples were processed and analyzed. The percent of FBA-TPQ that was metabolized by Phase I and Phase II enzymes was calculated by comparing the initial concentration of the iminoquinone to the measured concentration following incubation.

### 3.13. Animals

#### 3.13.1. Mouse Xenograft Model of Human Pancreatic Cancer

The animal use and care protocol was approved by the Institutional Animal Use and Care Committee of Texas Tech University Health Sciences Center. Female athymic pathogen-free nude mice (nu/nu, 4–6 weeks) were purchased from the Charles River Laboratories International. Inc. (Wilmington, MA, USA). To establish Panc-1 human pancreatic cancer xenograft tumors, cultured Panc-1 cells were harvested from monolayer cultures, washed twice with serum-free medium, re-suspended and injected sc (5 × 10^6^ cells, total volume 0.2 mL) into the left inguinal area of the mice. All animals were monitored for activity, physical condition, body weight, and tumor growth. Tumor size was determined every other day by caliper measurement of two perpendicular diameters of the implant. Tumor mass (in g) was calculated by the formula, 1/2*a* × *b*^2^ where “*a*” is the long diameter and “*b*” is the short diameter (in cm).

The animals bearing human cancer xenografts were randomly divided into various treatment groups and a control group (7–10 mice/group). The untreated control group received the vehicle only. For the Panc-1 xenograft model, FBA-TPQ was dissolved in PEG400: ethanol: saline (57.1: 14.3: 28.6, v/v/v),and was administered by intraperitoneal (ip) injection at doses of 5 and 10 mg/kg/d, 5 d/wk for 3 weeks. 

#### 3.13.2. Pharmacokinetic Studies in Mice

Female BALB/c (18–20 g) mice were purchased from Harlan Laboratories (Indianapolis, IN). All animals were fed with commercial diet and water *ad libitum* and were on an alternating 12 h light/dark cycle. The mice were randomly divided into groups of three, and were dosed either intravenously (5 mg/kg) or intraperitoneally (10 mg/kg) with FBA-TPQ in PEG 400: ethanol: saline (57.1%:14.3%:28.6%). Before dosing, and at 5, 15, 30, and 60 min, and 2, 4, 8, and 24 h after dosing, groups of animals (three/time point) were anesthetized, and blood was collected from the retro-orbital sinus into heparinized tubes. Plasma was obtained by centrifuging the blood samples at 14,000 rpm for 15 min. FBA-TPQ was then extracted from plasma and stored at −80 °C until analysis. The brain, heart, kidneys, liver, lungs, and spleen were also collected at the same time points during necropsy, blotted on Whatman No. 1 filter paper, trimmed of extraneous fat and connective tissue, weighed, and homogenized in phosphate-buffered saline. The resulting homogenates were stored at −80 °C until further processing and analysis. 

The average FBA-TPQ concentrations in plasma and tissues at each of the time points following intravenous and intraperitoneal administrations were used for pharmacokinetic analyses using a one- or two-compartmental model, respectively. The following pharmacokinetic parameters were estimated using Phoenix WinNonlin 6.0 (Mountain View, CA, USA): area under the concentration-time curve (AUC, h·μg/mL), half-life (T_½_, h), maximum concentration (C_max_, μg/mL), time of the maximum concentration (T_max_, h), and clearance (CL, mL/h/kg), as applicable. Bioavailability (*X*) was calculated as *X* = [AUC_ip_ × dose_iv_]/[AUC_iv_ × dose_ip_].

### 3.14. Data and Statistical Analysis

Experimental data are expressed as means and standard deviations, and the significance of differences was analyzed by ANOVA or Student’s *t*-test, as appropriate. 

## 4. Conclusions

The present study was the first to systematically investigate the novel synthetic makaluvamine analog FBA-TPQ’s *in vitro* and *in vivo* anti-tumor effects in pancreatic cancer cells. We observed that FBA-TPQ significantly decreased pancreatic cancer cell growth, induced cell apoptosis and led to cell cycle arrest. In a dose-dependent manner, FBA-TPQ decreased the growth of Panc-1 xenograft tumors with minimal host toxicity. To facilitate preclinical and clinical development of the compound, a RRLC-UV method was developed and validated for the quantification of FBA-TPQ. The method offered the advantages of high selectivity and simple sample preparation. It was successfully applied for the evaluation of the pharmacokinetics of FBA-TPQ in mice, making this the first report of the pharmacokinetics of FBA-TPQ. In *in vitro* assays, FBA-TPQ was stable in plasma, bound to protein extensively, and metabolized by S9 enzymes. It had a short plasma half-life and a wide tissue distribution in BALB/c mice. The results provide a basis for further preclinical and clinical studies of the compound as a candidate anti-cancer agent. 
